# Doxycycline Attenuates Leptospira-Induced IL-1β by Suppressing NLRP3 Inflammasome Priming

**DOI:** 10.3389/fimmu.2017.00857

**Published:** 2017-07-24

**Authors:** Wenlong Zhang, Xufeng Xie, Dianjun Wu, Xuemin Jin, Runxia Liu, Xiaoyu Hu, Yunhe Fu, Zhuang Ding, Naisheng Zhang, Yongguo Cao

**Affiliations:** ^1^Key Laboratory for Zoonosis Research, Ministry of Education, College of Veterinary Medicine, Jilin University, Changchun, China; ^2^Department of Clinical Veterinary Medicine, College of Veterinary Medicine, Jilin University, Changchun, China; ^3^South Dakota State University, Brookings, SD, United States; ^4^Department of Infectious Disease, College of Veterinary Medicine, Jilin University, Changchun, China

**Keywords:** doxycycline, *Leptospira*, IL-1β, NLRP3, caspase-1

## Abstract

Doxycycline (Dox), a semisynthetic antibiotic, has been reported to exert multiple immunomodulatory effects. Treatment with Dox has a satisfactory curative effect against leptospirosis. In addition to its antibacterial action, we supposed that Dox also modulated immune response in controlling leptospira infection. Using J774A.1 mouse macrophages, the effects of Dox on protein and mRNA levels of IL-1β and TNF-α were investigated after infection with live or sonicated *Leptospira interrogans* serovar Lai strain Lai (56601). Specifically, the level of IL-1β but not TNF-α was sharply decreased when treated with Dox in leptospira-infected macrophages. Western blot analysis showed that Dox suppressed the activation of leptospira-induced MAPK and NF-κB signaling pathways. Using NLRP3-deficient and NLRC4-deficient mice, the data showed that the expression of leptospira-induced IL-1β was mainly dependent on the presence of NLRP3 inflammasome in macrophages. Meanwhile, Dox suppressed leptospira-induced NLRP3 inflammasome priming with the upregulation of the Na/K-ATPase Pump β1 subunit. The inhibition effect of Dox on IL-1β was also conspicuous in cells with lipopolysaccharide and ATP stimulation. These results were confirmed *in vivo*, as peritoneal fluids of mice and organs of hamsters expressed less IL-1β after treatment of leptospiral infection with Dox. Our results indicated that Dox also modulated immune response to attenuate leptospira-induced IL-1β by suppressing p38, JNK, p65, and NLRP3 inflammasome priming.

## Introduction

Leptospirosis is a globally spread zoonotic disease caused by the virulent leptospira (1). In recent years, leptospirosis re-emergently outbreaks and is a relatively ignored disease especially in tropical countries. Transmission of the leptospires to humans occurs via direct contact with infected animals or by ingesting soil or water contaminated with leptospira (2). Severity ranges from a mild illness to a severe infection, and even death (3). Because of non-specific presentation and poor diagnostics (4), treatment with antibiotics against leptospirosis in time will improve survival rate. In the recent decade, the efficacy of doxycycline (Dox) against leptospirosis has been approved. It is unknown whether Dox also modulates immune response in treatment of leptospira infection.

Many effective therapeutic agents exhibit effects that are different from their intended primary mode of action (5), such as Dox, which suppressed inflammatory cytokine expression in a variety of experimental animal and cell models (5–8). Because leptospira induces severe inflammation in susceptible animals and humans, it is possible that Dox exerts both antibacterial and anti-inflammatory actions to lead to a satisfactory curative effect against leptospirosis. Unfortunately, the immunomodulatory effects and the detailed mechanisms of Dox in leptospira infection remain unclear.

Pattern recognition receptors, including the toll-like receptor (TLR) and the NOD-like receptor (NLR), can recognize leptospira. Stimulations TLR2 and TLR4 by leptospira result in activation of the NF-κB and MAPK signaling pathways, which are involved in transcriptional induction of many proinflammatory genes (2, 9). Leptospira also stimulate NLRP3 inflammasome, the best characterized NLR molecules, inducing the activation of caspase-1 and the release of mature IL-1β (10). It is reported that the leptospira lipopolysaccharide (LPS) provided the first signals for inflammasome priming and the leptospiral glycolipoprotein activated inflammasome (10). It is unknown whether other inflammasomes are stimulated by leptospira, for example, NLRC4. The detailed pathogenesis of leptospira is unknown, which limits the development of the ideal treatment against leptospirosis.

The excessive production of proinflammatory agents will cause pathological inflammatory disorders and tissue injury (11), which is why some studies paid close attention to immunosuppressive agents in leptospirosis (12, 13). In this study, we sought to examine the immunomodulatory effects of Dox in leptospira-infected macrophages of mice. The results showed that Dox attenuate leptospira-induced IL-1β by suppressing p38, JNK, p65, and NLRP3 inflammasome priming. Our study also provides a possible therapeutic value with antibiotics and immunomodulators against leptospirosis.

## Materials and Methods

### Bacterial Strain and Animals

Pathogenic *Leptospira interrogans* serovar Lai strain Lai (56601) was grown in liquid Ellinghausen–McCullough–Johnson–Harris (EMJH) medium at 29°C. The virulence of the leptospira was maintained by passage in hamster. Before use, bacterial concentration was determined using a Petroff–Hausser counting chamber. Leptospira was passaged less than three times in liquid EMJH for all infection studies. C57BL/6J wild-type (WT) mice were provided by the Model Animal Research Center of Nanjing University. C57BL/6J NLRP3-dificient (NLRP3^−/−^) and NLRC4-dificient (NLRC4^−/−^) mice were kindly provided by Dr. Feng Shao. Syrian golden hamsters (*Mesocricetus auratus*) were provided by the Animal Center of Jilin University. All animal experiments were performed according to regulations of the Administration of Affairs Concerning Experimental Animals in China. The protocol was approved by the Institutional Animal Care and Use Committee of Jilin University (20170318).

### Cell Culture

The extraction and culture of primary mouse macrophages were completed as previously described (14). In brief, WT, NLRP3^−/−^, and NLRC4^−/−^ mice were injected with 2 ml of 3% thioglycolate medium (BD Biosciences, USA). Three days after the injection, peritoneal macrophages were isolated by washing the peritoneal cavity with PBS. The primary mouse macrophages and murine macrophage cell line J774A.1 were cultured in DMEM media (HyClone, USA) supplemented with 10% fetal bovine serum (FBS; HyClone, USA), 100 U/ml penicillin and 100 µg/ml streptomycin in 5% CO_2_ atmosphere at 37°C. Before infection, cells were washed three times by DMEM media to dislodge the penicillin and streptomycin.

### Effect of Dox on Cell Viability

J774A.1 cells at a density of 10^3^ were incubated with Dox (0–20 µg/ml) for 24 and 48 h at 37°C in a 96-well cell culture plate, followed by the addition of 10 μl/well CCK-8 (TransGen Biotech, China). After incubated for 2 h at 37°C, the optical density of the samples at 450 nm was measured.

### Experimental Infections and Treatment *In Vitro*

J774A.1 cells and primary mouse macrophages were seeded at 10^6^ cells in six-well culture plates and pre-incubated for 12 h in DMEM media supplemented with 10% FBS. In all experiments, unless otherwise noted, macrophages were incubated in the presence or absence of various concentrations of Dox (Sigma-Aldrich, USA) added 24 h prior to 10^8^ live or sonicated leptospira infection (MOI of 100:1) (7, 15). The cells were then washed by culture medium and incubated for another 24 h with live or sonicated leptospira alone or with added various concentrations of Dox (15). To activate NLRP3 inflammasome, J774A.1 cells were incubated in the presence or absence of Dox added 24 h prior to stimulate with LPS (1 µg/ml) from Escherichia coli 0111:B4 (Sigma-Aldrich, USA) for 4 h. The cells were then washed by culture medium and incubated for 2 h with ATP (5 mM) (Sigma-Aldrich, USA) alone or with added Dox. To distinguish priming from activating, J774A.1 cells were primed with LPS (1 µg/ml) for 4 h, and then incubated with Dox for 1 h before being pulsed with ATP (5 mM) for 2 h. To know how long these effects last once Dox has been removed, J774A.1 cells were incubated with Dox (20 µg/ml) for 24 h. After 0–4 h once Dox has been removed, the cells were stimulated with live leptospira for another 24 h without Dox.

### Experimental Infections and Treatment *In Vivo*

Mice were injected intraperitoneally with Dox at the dose of 5 mg/kg before 24 h inoculated intraperitoneally with 2 × 10^8^ sonicated leptospira. Another 24 h later, mice were euthanized and their peritoneal cavities were lavaged with 5 ml of PBS and centrifuged at 2,000 rpm for 10 min as in a previous study (16). The cytokine expressions of the supernatants (Sups) were analyzed by enzyme-linked immunosorbent assay (ELISA). To verify the immunoregulatory activity of Dox against acute leptospirosis, hamsters were inoculated intraperitoneally with 10^7^ live leptospira, which induced 100% mortality in hamsters (17). Dox at the dose of 5 mg/kg was performed intraperitoneally at 48 and 72 h after infection (18). At 96 h after infection, hamsters were euthanized and their organs were collected.

### Total RNA Isolation and Quantitative Real-Time Polymerase Chain Reaction (qRT-PCR)

Total RNA of cells and organs were extracted using TRIzol (Invitrogen, USA) following the manufacturer’s instructions. RNA was reverse transcribed into cDNA by using random primers from a TransScript One-Step gDNA Removal kit and cDNA Synthesis SuperMix (TransGen Biotech, China). The primers used in this study were listed in Table 1. The qPCR reaction was performed using an Applied Bioscience 7500 thermocycler and FastStart Universal SYBR Green Master (Roche Applied Science, Germany). PCR conditions were as follows: 50°C for 2 min, 95°C for 10 min, followed by 45 cycles of amplification 95°C for 15 s and 60°C for 60 s (14). The number of target gene was normalized to GAPDH using a 2^−ΔΔCT^ method.

**Table 1:** Sequence of primers used for quantitative polymerase chain reaction assays.

Gene	Primer	Sequence (5′–3′)
Mice GAPDH	Sense	AGGTCGGTGTGAACGGATTTG
	Anti-sense	GGGGTCGTTGATGGCAACA
Hamster GAPDH	Sense	GATGCTGGTGCCGAGTATGT
	Anti-sense	GCCACGCCCACATCATTC
Mice IL-1β	Sense	ACCTGTGTCTTTCCCGTGG
	Anti-sense	TCATCTCGGAGCCTGTAGTG
Hamster IL-1β	Sense	TTCTGTGACTCCTGGGATGGT
	Anti-sense	GTTGGTTTATGTTCTGTCCGTTG
Mice TNF-α	Sense	CCTATGTCTCAGCCTCTTCTCAT
	Anti-sense	CACTTGGTGGTTTGCTACGA
Hamster TNF-α	Sense	GGTGATACCAGCAGACGG
	Anti-sense	CTTGATGGCGGACAGGA
Mice NLRP3	Sense	CCCTTGGAGACACAGGACTC
	Anti-sense	GAGGCTGCAGTTGTCTAATTCC
Mice Na/Kβ1	Sense	CTGCCTGGCTGGCATCTT
	Anti-sense	TGGTATGTGGGCTTCAGTTCAC

### Enzyme-Linked Immunosorbent Assay

The suspensions of J774A.1 cells in six-well culture plates and the peritoneal cavities of mice were centrifuged at 2,000 rpm for 10 min at 4°C, then stored at −80°C. Cytokines were measured in Sups subsequently using mouse ELISA kits (eBioscience, USA) according to the instructions of the manufacturer. Three biological replicates were carried out.

### Lactate Dehydrogenase (LDH) Release

J774A.1 cells at a density of 10^4^ were incubated in the presence or absence of Dox in 96-well culture plates for 24 h. Then cells were stimulated by live/dead leptospira and LPS/ATP with the presence or absence of Dox for another 24 h. The LDH release was tested by a Cytotoxicity Detection Kit (Roche Applied Science, Germany) according to the instructions of the manufacturer.

### Western Blot Analysis

Total proteins of cells were extracted using M-PER Mammalian Protein Extraction Reagent (Thermo Scientific, USA). Protein concentrations were determined by Pierce BCA protein assay kit (Thermo Scientific, USA). Protein samples (50 μg/well) were separated by sodium dodecyl sulfate–polyacrylamide gel electrophoresis and then transferred onto the polyvinylidene difluoride membrane. After blocked 2 h at room temperature with 5% skim milk dissolved in Tris buffer solution containing 0.05% Tween-20 (TBS-T), and followed by washing with TBS-T, the membrane was incubated with primary antibodies overnight at 4°C. Subsequently, the membrane was washed and then incubated with secondary antibody at room temperature for 2 h. After washed in TBS-T three times, membranes were tested by ECL Plus Western Blotting Detection System (Amersham Life Science, UK). The primary antibodies used were rabbit monoclonal antibodies, and the secondary antibody used was HRP-conjugated goat anti-rabbit antibody (Cell Signaling Technology, Inc., USA). Antibodies p65, p-p65, p38, ERK, p-ERK, NLRP3, and IL-1β were purchased from Cell Signaling Technology, Inc. in USA. Antibody p-p38 was purchased from ABclonal in USA. Antibody β-actin was purchased from Bioworld in USA and caspase-1 was from Abcam in USA. The gray values of the western blotting bands were procured using Image-Pro Plus 6.0 software.

### Statistical Analysis

Data are presented as mean ± SD. Statistical analyses were performed by One-way ANOVA followed by the Newman–Keuls test. Differences were considered significant at *p* < 0.05.

## Results

### Dox Inhibited Leptospira-Induced IL-1β mRNA and Protein Levels in Macrophages of Mice

**Figure 1:**
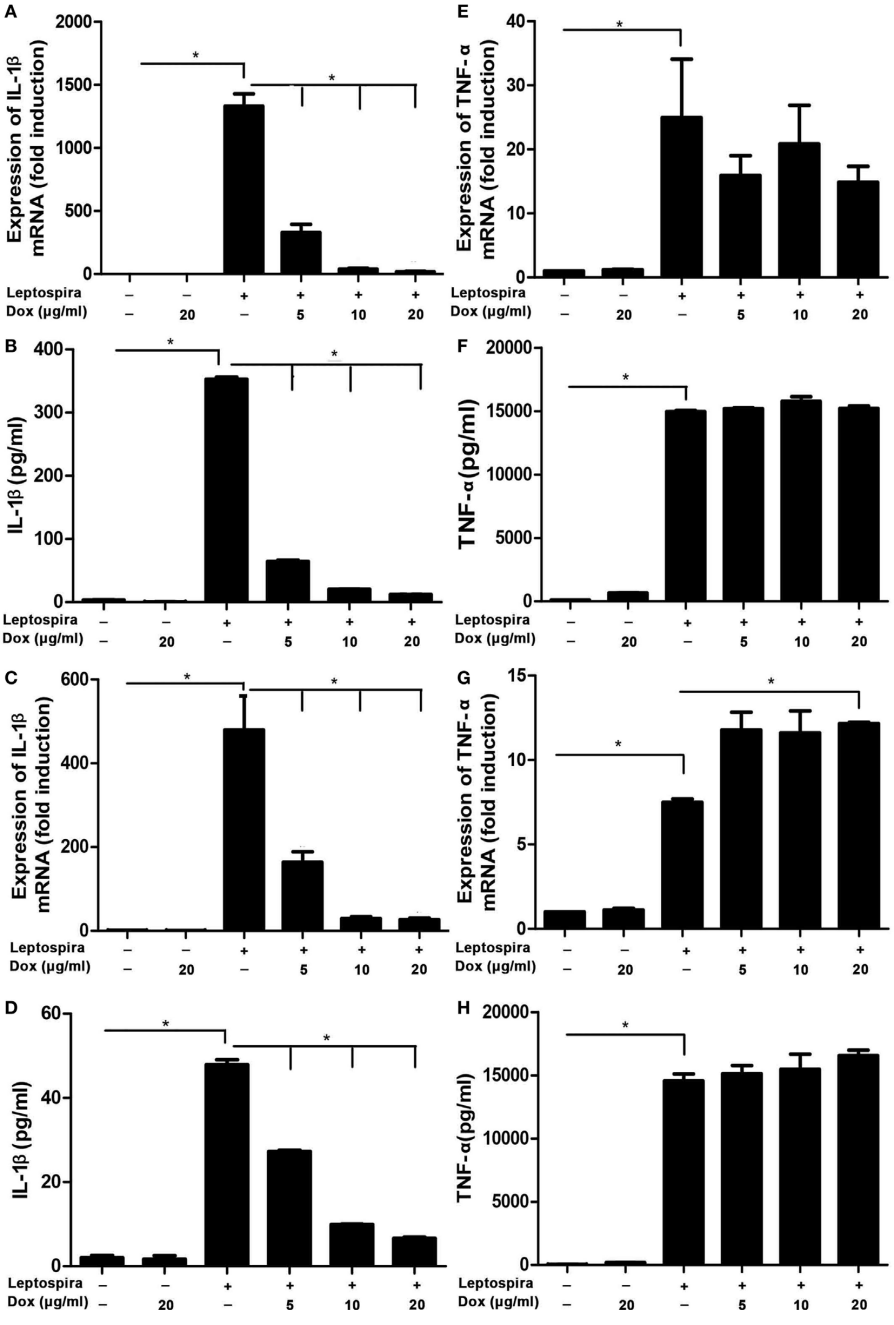
Doxycycline inhibited leptospira-induced IL-1β mRNA and protein levels in J774A.1 cells. The level of mRNA of IL-1β and TNF-α was analyzed by quantitative real-time polymerase chain reaction and the protein level in supernatant (Sup) was analyzed by enzyme-linked immunosorbent assay (ELISA). **(A,B,E,F)** Cells were infected by live leptospira. **(C,D,G,H)** Cells were infected by sonicated leptospira. The mRNA levels of IL-1β **(A,C)** and TNF-α **(E,G)** were normalized to the expression of the housekeeping gene GAPDH. The mRNA levels of cytokines in untreated controls were set as 1.0. The protein levels of IL-1β **(B,D)**, and TNF-α **(F,H)** in Sup were measured by ELISA. Bars show the levels of cytokines with mean ± SD (*n* = 3) and analyzed by the one-way ANOVA. *p < 0.05.

To analyze the potential anti-inflammatory effects of Dox, IL-1β, and TNF-α, mRNA were analyzed by qRT-PCR, protein levels in Sup were tested by ELISA in leptospira-infected J774A.1 cells. Dox at the doses of 5, 10, and 20 µg/ml was not cytotoxic to J774A.1 cells for 24 and 48 h (Figure S1 in Supplementary Material). The live leptospira-induced high expressions and releases of IL-1β and TNF-α compared with the untreated control group (Figures 1A,B,E,F). Interestingly, after treatment with different concentrations of Dox, only the IL-1β expression and release were reduced dramatically in leptospira-infected J774A.1 cells (Figures 1A,B). In order to eliminate the influence of antibiotic sterilization function on live leptospira, this experiment was repeated using sonicated leptospira. The sonicated leptospira also induced high expressions and releases of IL-1β and TNF-α (Figures 1C,D,G,H). Compared with the live leptospira, sonicated leptospira-induced less IL-1β mRNA expression and protein release (Figures 1A–D). Similarly, when Dox was treated, only the IL-1β expression and release were reduced in sonicated leptospira-infected J774A.1 cells (Figures 1C,D). These results showed that Dox specifically inhibited leptospira-induced IL-1β but not TNF-α mRNA and protein level in macrophages of mice.

### MAPK and NF-κB Pathways Were Downregulated in Macrophages Treated with Dox during Leptospira Infection

**Figure 2:**
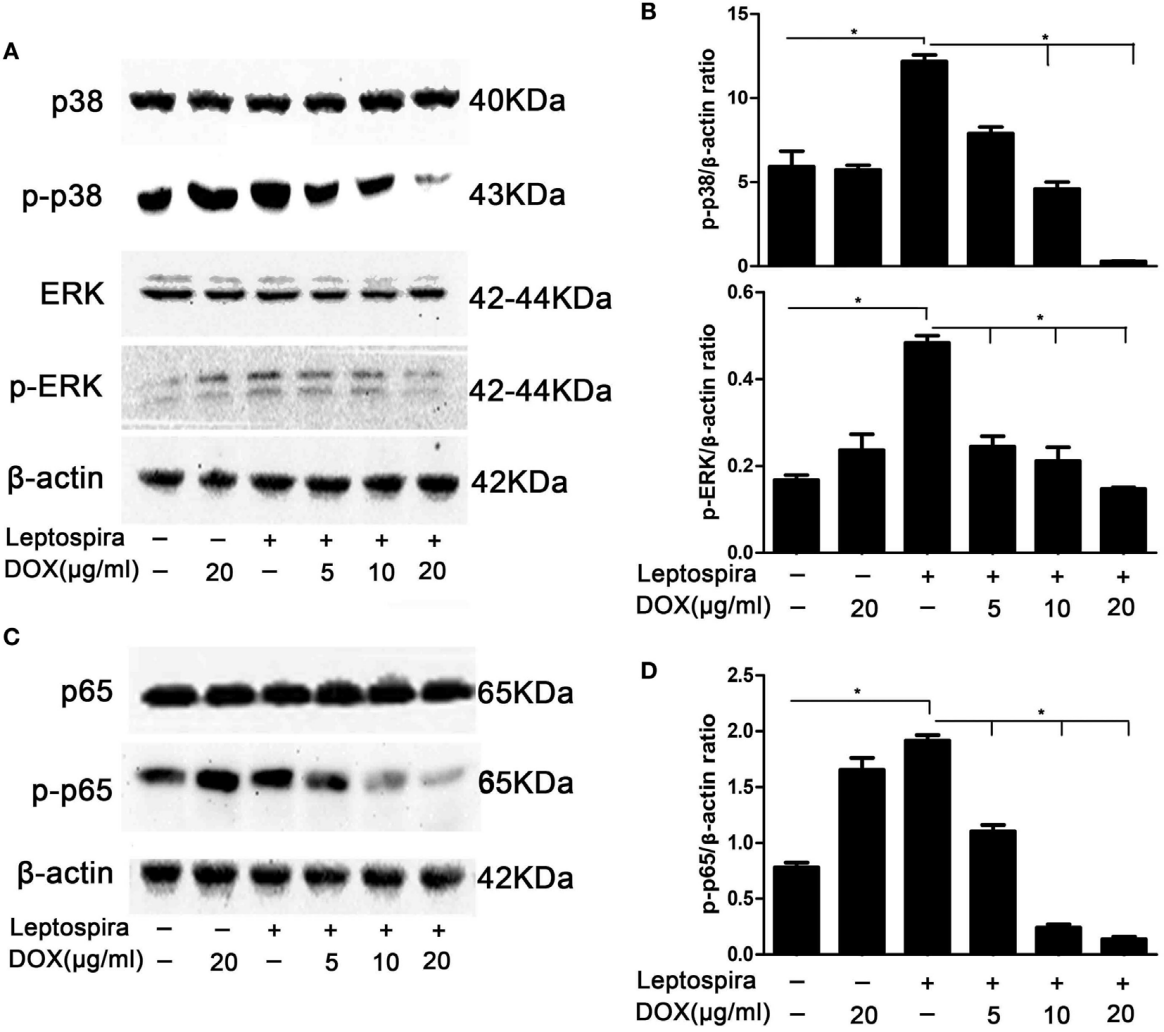
Doxycycline (Dox) inhibited the activation of MAPK and NF-κB pathways in sonicated leptospira-infected J774A.1 cells. J774A.1 cells were seeded at 10^6^ cells in six-well culture plates and macrophages were incubated in the presence or absence of various concentrations of Dox added 24 h prior to 10^8^ sonicated leptospira infection. The cells were washed in culture medium and incubated for another 24 h with sonicated leptospira alone or with added various concentrations of Dox. After that, the total proteins of cells were extracted and analyzed by western blot with specific antibodies. The β-Actin protein was used as a control. The activation of MAPK **(A)** and NF-κB **(C)** pathways was detected by immunoblot. The gray value of p-p38, p-ERK **(B)**, and p-p65 **(D)** was procured using Image-Pro Plus 6.0 software. Each column shows the mean ± SD of three independent experiments and analyzed by the one-way ANOVA. *p < 0.05.

MAPK and NF-κB pathways play critical roles in the induction of proinflammatory gene expression. To confirm whether the inhibition of IL-1β by Dox is mediated through the MAPK and NF-κB pathways, activation of p38, ERK, and p65 was examined by western blot in sonicated leptospira-infected J774A.1 cells. In the MAPK pathway, Dox inhibited the activation of p38 and ERK in sonicated leptospira-infected J774A.1 cells (Figures 2A,B). In the NF-κB pathway, Dox inhibited the activation of p65 (Figures 2C,D). The activation of p38, ERK, and p65 was inhibited by Dox in a dose-dependent manner. These results indicated that the inhibition of the downregulation of MAPK and NF-κB pathways by Dox in leptospira-infected J774A.1 cells may be responsible for the decline of IL-1β.

### Leptospira-Induced IL-1β Release Was Mainly Dependent on the NLRP3, Not the NLRC4 Inflammasome

**Figure 3:**
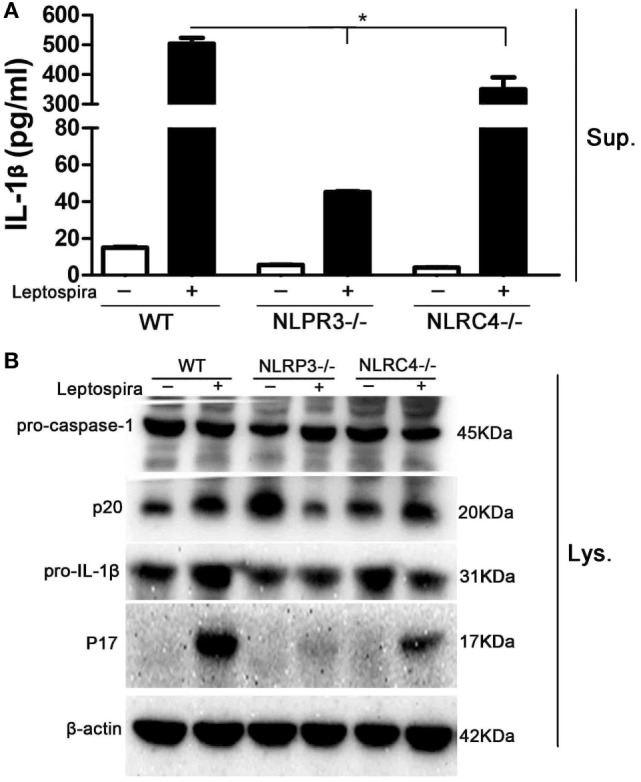
Leptospira-induced IL-1β release was mainly dependent on the NLRP3, not the NLRC4 inflammasome. Peritoneal macrophages were infected live leptospira at MOI of 100. **(A)** The protein levels of IL-1β in cell supernatants were measured by enzyme-linked immunosorbent assay. Bars show the levels of cytokines with mean ± SD (*n* = 3) and analyzed by the one-way ANOVA. *p < 0.05 vs. leptospira-infected group in wild-type mice. **(B)** The activation of caspase-1 and the release of mature IL-1β in peritoneal macrophages extracts (Lysate) were analyzed by immunoblot. The β-Actin protein was used as a control.

Both NLRP3 and NLRC4 inflammasomes were responsible for the expression of mature IL-1β. To make clear, the role of NLRP3 and NLRC4 inflammasomes in leptospira-induced IL-1β release, peritoneal macrophages from WT, NLRP3^−/−^, and NLRC4^−/−^ mice were used. The level of IL-1β release in Sup was decreased significantly in peritoneal macrophages of NLRP3^−/−^ and NLRC4^−/−^ mice after live leptospira infection compared with infected cells of WT mice (Figure 3A). Interestingly, the release of IL-1β was more seriously damaged in infected cells of NLRP3^−/−^ mice. Western blot analysis showed that the defect of NLRP3 damaged the activation of caspase-1 and the maturity of IL-1β (Figure 3B). These results indicated that leptospira-induced IL-1β release was mainly dependent on NLRP3 inflammasome activation.

### Dox Suppressed NLRP3 Inflammasome Priming with Upregulation of the Expression of the Na/Kβ1 Subunit during Leptospira Infection

**Figure 4:**
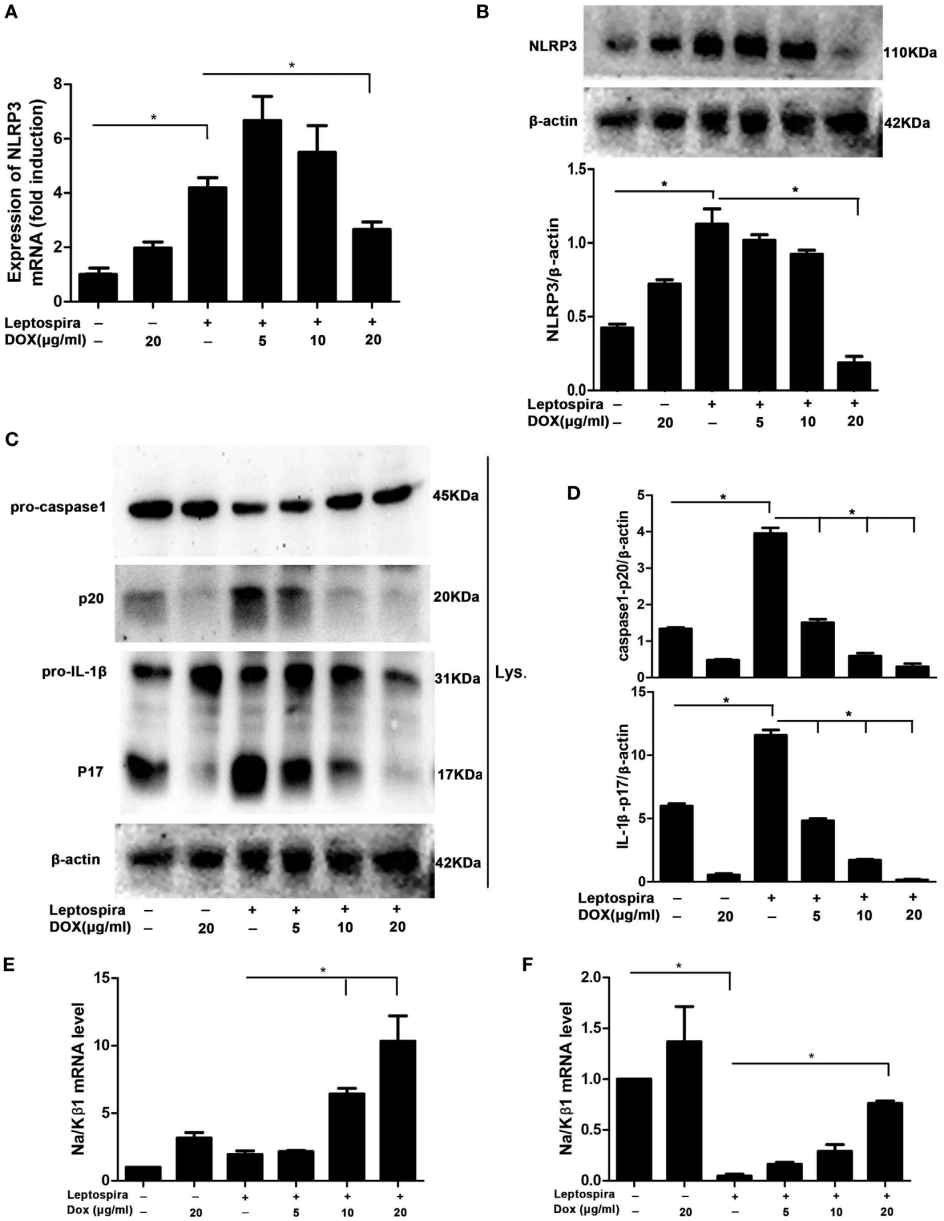
Doxycycline suppressed NLRP3 inflammasome priming with upregulation of the expression of the Na/Kβ1 subunit during leptospira infection. The expression of NLRP3 mRNA **(A)** in sonicated leptospira-infected J774A.1 cells was analyzed by quantitative real-time polymerase chain reaction (qRT-PCR). The mRNA levels of NLRP3 in J774A.1 cells were normalized to the expression of the housekeeping gene GAPDH. The expression of NLRP3 protein **(B)**, the activation of caspase-1, and the release of mature IL-1β in cell extracts (Lysate) **(C,D)** were analyzed by western blot. The β-Actin protein was used as a control. The expression of Na/Kβ1 subunit mRNA was analyzed by qRT-PCR in sonicated **(E)** and live **(F)** leptospira-infected J774A.1 cells. The mRNA levels of Na/Kβ1 subunit in J774A.1 cells were normalized to the expression of the housekeeping gene GAPDH. All bars show the mean ± SD of three independent experiments and analyzed by the one-way ANOVA. *p < 0.05.

As the NLRP3 inflammasome is vital to the release of mature IL-1β, we wanted to make clear whether the inhibition of IL-1β by Dox is also mediated through the NLRP3 inflammasome. The NLRP3 mRNA and protein were analyzed by qRT-PCR and western blot, respectively, in sonicated leptospira-infected J774A.1 cells. Leptospira induced the high expression of NLRP3 in both mRNA and protein levels. After treatment with the Dox at a dose of 20 µg/ml, the expression of leptospira-induced NLRP3 was declining (Figures 4A,B). The activation of caspase-1 and the release of mature IL-1β were also analyzed by western blot. As shown in Figures 4C,D, Dox inhibited the leptospira-induced activation of caspase-1 and the release of mature IL-1β. These results indicated that Dox suppressed NLRP3 inflammasome priming during leptospira infection.

Leptospira can activate the NLRP3 inflammasome by downregulation of the Na/K-ATPase pump. The Na/K-ATPase pump is composed of Na/Kα and Na/Kβ1 subunits (10). It is reported that leptospira activated the NLRP3 inflammasome by downregulating the expression of the Na/Kβ1 subunit (10). To make clear whether Dox upregulated the expression of the Na/Kβ1 subunit in leptospira-infected macrophages, the Na/Kβ1 subunit mRNA was analyzed by qRT-PCR. After infection of sonicated leptospira, the expression of the Na/Kβ1 subunit was not downregulated (Figure 4E). Then we used live leptospira to repeat this experiment and the markedly downregulated Na/Kβ1 subunit was found (Figure 4F). Dox upregulated the expression of the Na/Kβ1 subunit in both sonicated and live leptospira-infected macrophages (Figures 4E,F). These results indicated that Dox suppressed NLRP3 inflammasome possibly by upregulating the expression of the Na/Kβ1 subunit in leptospira-infected macrophages.

### The Suppression Effect of Dox to NLRP3 Inflammasome Was Not Limited to Leptospira Stimulation

**Figure 5:**
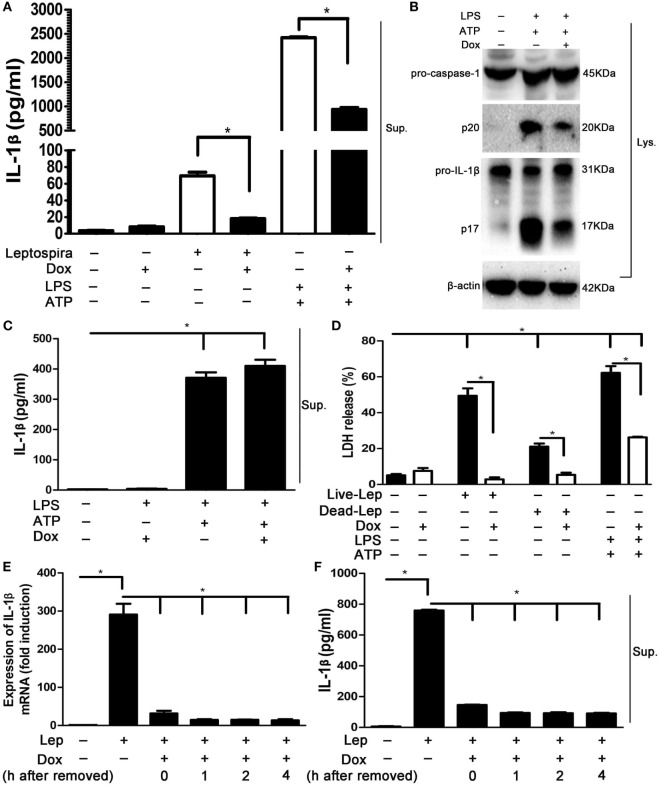
The suppression effect of doxycycline to NLRP3 inflammasome was not limited to leptospira stimulation. **(A)** The protein levels of IL-1β in cell supernatants (Sups) were measured by enzyme-linked immunosorbent assay (ELISA). **(B)** The activation of caspase-1 and the release of mature IL-1β in J774A.1 cells extracts (Lysate) were analyzed by immunoblot. The β-Actin protein was used as a control. **(C)** The releases of IL-1β in lipopolysaccharide-primed J774A.1 cells Sups were measured by ELISA. **(D)** The level of cell death was assessed by measuring release of lactate dehydrogenase. **(E)** The expression of IL-1β mRNA in live leptospira-infected J774A.1 cells was analyzed by quantitative real-time polymerase chain reaction. The mRNA levels of IL-1β in J774A.1 cells were normalized to the expression of the housekeeping gene GAPDH. **(F)** The releases of IL-1β in live leptospira-infected J774A.1 cells Sups were measured by ELISA. Bars show the levels of cytokines with mean ± SD (*n* = 3) and analyzed by the one-way ANOVA. *p < 0.05.

To determine whether Dox-mediated NLRP3 inflammasome inhibition is specific to leptospira stimulation, the effects of Dox on LPS and ATP stimulation, a conventional NLRP3 inflammasome agonist (16), was assessed. The LPS and ATP stimulation induced massive expression of IL-1β, which was stronger than sonicated leptospira infection in macrophages. Interestingly, treatment with Dox inhibited the expression of IL-1β in both leptospira and agonist stimulation (Figure 5A). As expected, Dox suppressed the activation of caspase-1 and the release of mature IL-1β in LPS and ATP stimulated macrophages by western blot analysis (Figure 5B). These results indicated that Dox-mediated NLRP3 inflammasome inhibition was not limited to leptospira stimulation. To distinguish priming from activating, LPS-primed J774A.1 cells were incubated with Dox (20 µg/ml) for 1 h, followed by treatment with ATP for 2 h. The releases of IL-1β in Sup were tested by ELISA. The level of IL-1β release was still high after Dox treated (Figure 5C). So Dox is only affecting priming of NLRP3 inflammasome. The effect of Dox on leptospira- and agonist-induced cell death was studied by LDH release. As expected, pretreatment with Dox dramatically reduced the level of cell death induced by leptospira and agonist (Figure 5D). To study how long these effects last once Dox has been removed, J774A.1 cells were incubated with Dox (20 µg/ml) for 24 h. After 0–4 h once Dox has been removed, the cells were stimulated with live leptospira for another 24 h without Dox. The expression and release of IL-1β was tested by qRT-PCR and western blot, respectively. The data showed that the inhibitory effect of Dox persisted at least 4 h after Dox had been removed (Figures 5E,F).

### Dox Downregulated Leptospira-Induced IL-1β in Mice and Hamsters

**Figure 6:**
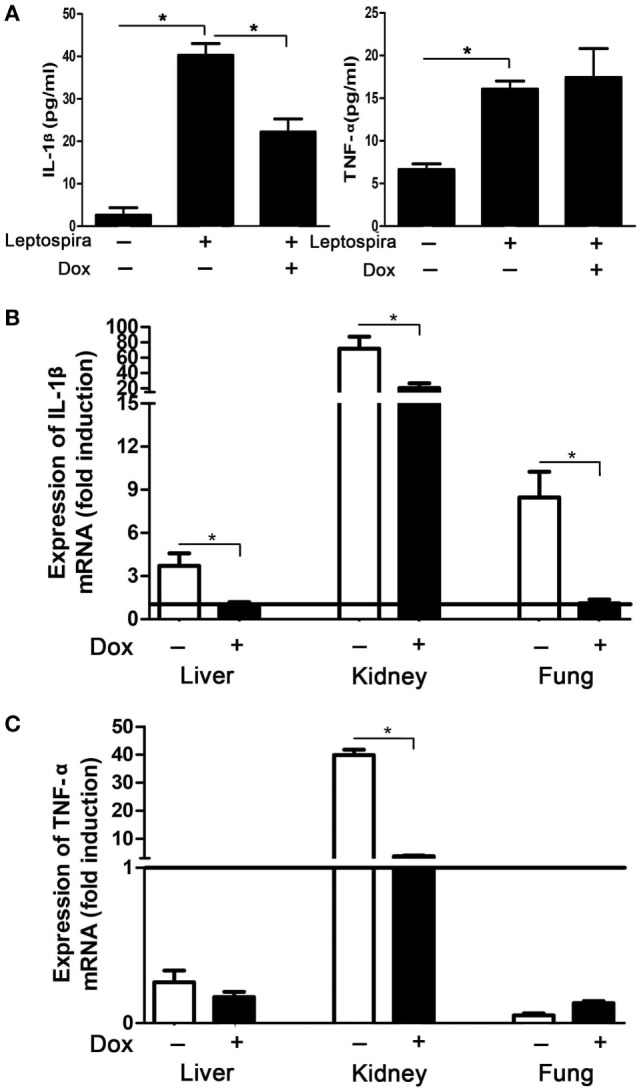
Doxycycline (Dox) downregulated leptospira-induced IL-1β in mice and hamsters. **(A)** Mice were injected intraperitoneally with Dox before 24 h inoculated intraperitoneally with 2 × 10^8^ sonicated leptospira. Another 24 h later, the peritoneal cavities were lavaged and the IL-1β and TNF-α were analyzed by enzyme-linked immunosorbent assay. Bars show the mean ± SD of three independent experiments and analyzed by the one-way ANOVA. *p < 0.05. **(B,C)** Hamsters were infected 10^7^ live leptospira and treated with Dox. At the 96 h after infection, the mRNA expression of IL-1β **(B)** and TNF-α **(C)** were analyzed by quantitative real-time polymerase chain reaction. Results were normalized to the expression of the housekeeping gene GAPDH. The mRNA levels of cytokines in untreated controls were set as 1.0 (solid line). Bars show the mean ± SD of three independent experiments and analyzed by the one-way ANOVA. *p < 0.05.

To verify the suppression effect of Dox on leptospira-induced IL-1β *in vivo*, mice were injected intraperitoneally with Dox before 24 h inoculated intraperitoneally with 2 × 10^8^ sonicated leptospira. Another 24 h later, the peritoneal cavities were lavaged and the cytokines were analyzed by ELISA. The expression of IL-1β and TNF-α was improved with leptospira stimulation compared with untreated controls. After treatment with Dox, only IL-1β levels were downregulated (Figure 6A). This was consistent to the results *in vitro*. To make clear whether Dox exerted the immunoregulatory activity against acute leptospirosis, hamsters were infected leptospira and treated with Dox. At 96 h after infection, the mRNA expression of IL-1β was downregulated in all the organs (liver, kidney, and lung) (Figure 6B), while TNF-α was downregulated only in the kidney (Figure 6C). These results indicated that Dox suppressed leptospira-induced IL-1β *in vivo*.

## Discussion

Leptospirosis, as a widespread zoonotic disease, causes a variety of symptoms, which range from a mild illness to a severe infection and even death (3). Many antimicrobial agents are active against multiple leptospira serovars *in vitro* (19, 20). However, the efficacy of most antibiotics is not better than Dox *in vivo* (4, 21, 22), and some antibiotics worsen leptospirosis when low doses are administered (23). Infected animals are expected to perish soon after showing clinical signs of infection, even if some antibiotics are available for treatment. Finding the anti-leptospira mechanism of Dox will contribute to the development of new therapies against leptospirosis. This study showed the immunoregulatory activity of Dox in leptospira-infected macrophages and *in vivo*. It may contribute to a better understanding of the anti-leptospira mechanism of Dox.

Massive overexpression of proinflammatory mediators was associated with a poor outcome in leptospirosis (24). Study reported that treatment with immunosuppressive agents alleviated the pathology of leptospirosis (12). In this study, we found that Dox particularity suppressed IL-1β expression, while the TNF-α expression was not declining after treatment with Dox (Figure 1). The following western blot showed that the inhibition of the phosphorylation of p38, JNK, and p65 by Dox may be responsible for the decline of IL-1β (Figure 2). Another study reported that Dox decreased iNOS expression by decreasing the p38 MAPK protein as well (5). It seems that the p38 pathway may be a target of Dox to exert immunomodulatory effects. The NF-κB and MAPK signal pathways were inhibited by Dox in leptospira-infected cell at 24 h. Though IL-1β and TNF-α are NF-κB-dependent genes, the expression of TNF-α was not decreasing as the IL-1β after Dox treated. These findings indicate that the expression of TNF-α induced by leptospira may via NF-κB- and MAPK-independent pathway.

It is unknown whether the NLRC4 inflammasome was responsible to leptospira-induced IL-1β expression. Our results indicated that a few of IL-1β could be induced by leptospira via NLRC4 inflammasome, while a large proportion of IL-1β was induced via NLRP3 inflammasome (Figure 3). Although some studies researched the relationship of NLRP3 inflammasome and leptospira (10, 25), the role of NLRP3 inflammasome in the process of leptospirosis is still unclear. A previous study reported that expression of NLRP3 was induced in stable transfectants by Dox (26). In our study, the expression of NLRP3 was improved by treatment with single Dox in both mRNA and protein levels compared with the untreated control (Figures 4A,B). The expression of NLRP3 was improved by the treatment with sonicated leptospira. However, contrasted with the treatment with single leptospira, the expression of the NLRP3 protein was reduced when Dox (20 µg/ml) was added (Figure 4B).

We showed that Dox inhibited the leptospira-induced activation of caspase-1 and the release of mature IL-1β in a dose-dependent manner (Figures 4C,D). Single Dox also inhibited the activation of caspase-1 and the expression of mature IL-1β. Although single Dox improved the expression of NLRP3, the upregulated expression of the Na/Kβ1 subunit by Dox inhibited the activation of NLRP3 inflammasome (Figures 4E,F). We found that the downregulation of the Na/K-ATPase β1 subunit was presented only after the live but sonicated leptospira infection. However, previous study reported that both the live and heat-killed leptospira infection downregulated the expression of the β1 subunit of the Na/K-ATPase in BMDMs (10). We speculated the ultrasonication of leptospira might break leptospiral glycolipoprotein, the inhibitor of Na/Kβ1 subunit, so that sonicated leptospira failed to downregulate the Na/K-ATPase β1 subunit. Although the expression of Na/Kβ1 was not downregulated after sonicated leptospira infection compared with the untreated control, the activation of NLRP3 inflammasome was still increased. These findings suggest that the downregulation of Na/K-ATPase β1 subunit were not necessary to activate the NLRP3 inflammasome in leptospira-infected macrophages. But the upregulation of the Na/Kβ1 subunit by Dox during leptospira infection inhibited the activation of NLRP3 inflammasome.

To our best knowledge, our work is the first to show that Dox downregulated IL-1β by suppressing NLRP3 inflammasome activation. And this suppression effect was not limited to leptospira stimulation. It also included a conventional NLRP3 inflammasome agonist, LPS, and ATP. Using mice and hamsters, we showed that Dox suppressed leptospira-induced IL-1β *in vivo*. As the efficacy of Dox against leptospirosis is acceptable, the inhibition of IL-1β levels may be a new treatment strategy against leptospirosis.

In this study, the immunoregulatory activity of Dox was investigated in leptospira-infected macrophages and *in vivo*. We showed that treatment with Dox dramatically inhibited the expression of leptospira-induced IL-1β. Western blot analysis indicated that Dox attenuated leptospira-induced IL-1β by suppressing MAPK, NF-κB, and NLRP3 inflammasome activation. The present findings may explain why Dox is more effective against leptospirosis than others. This study also provides the possible therapeutic value with antibiotics and immunomodulators against leptospirosis.

## Ethics Statement

All animal experiments were performed according to regulations of the Administration of Affairs Concerning Experimental Animals in China. The protocol was approved by the Institutional Animal Care and Use Committee of Jilin University (20170318).

## Author Contributions

Conceptualization: YC, WZ. Data curation: NZ. Formal analysis: WZ. Funding acquisition: YC, YF, ZD. Investigation: XJ. Methodology: XX, XJ, XH, DW. Project administration: YC, YF, ZD. Software: XX, WZ. Supervision: YC, NZ. Validation: WZ. Visualization: WZ. Writing: WZ, XX, RL.

## Conflict of Interest Statement

The authors declare that the research was conducted in the absence of any commercial or financial relationships that could be construed as a potential conflict of interest.

## Supplementary Material

Figure S1 Effect of doxycycline on the cell viability of J774A.1 cells. Cells were cultured with different concentrations of doxycycline (0–20 µg/ml) for 24 and 48 h. The cell viability was determined by CCK-8 kit. Bars show the mean ± SD of three independent experiments.
