# Supplementary figures and images for: Doxycycline Attenuates Leptospira-Induced IL-1β by Suppressing NLRP3 Inflammasome Priming
Source: Front Immunol. 2017 Jul 24;8:857. doi: 10.3389/fimmu.2017.00857 (PMC5522854; doi:10.3389/fimmu.2017.00857)

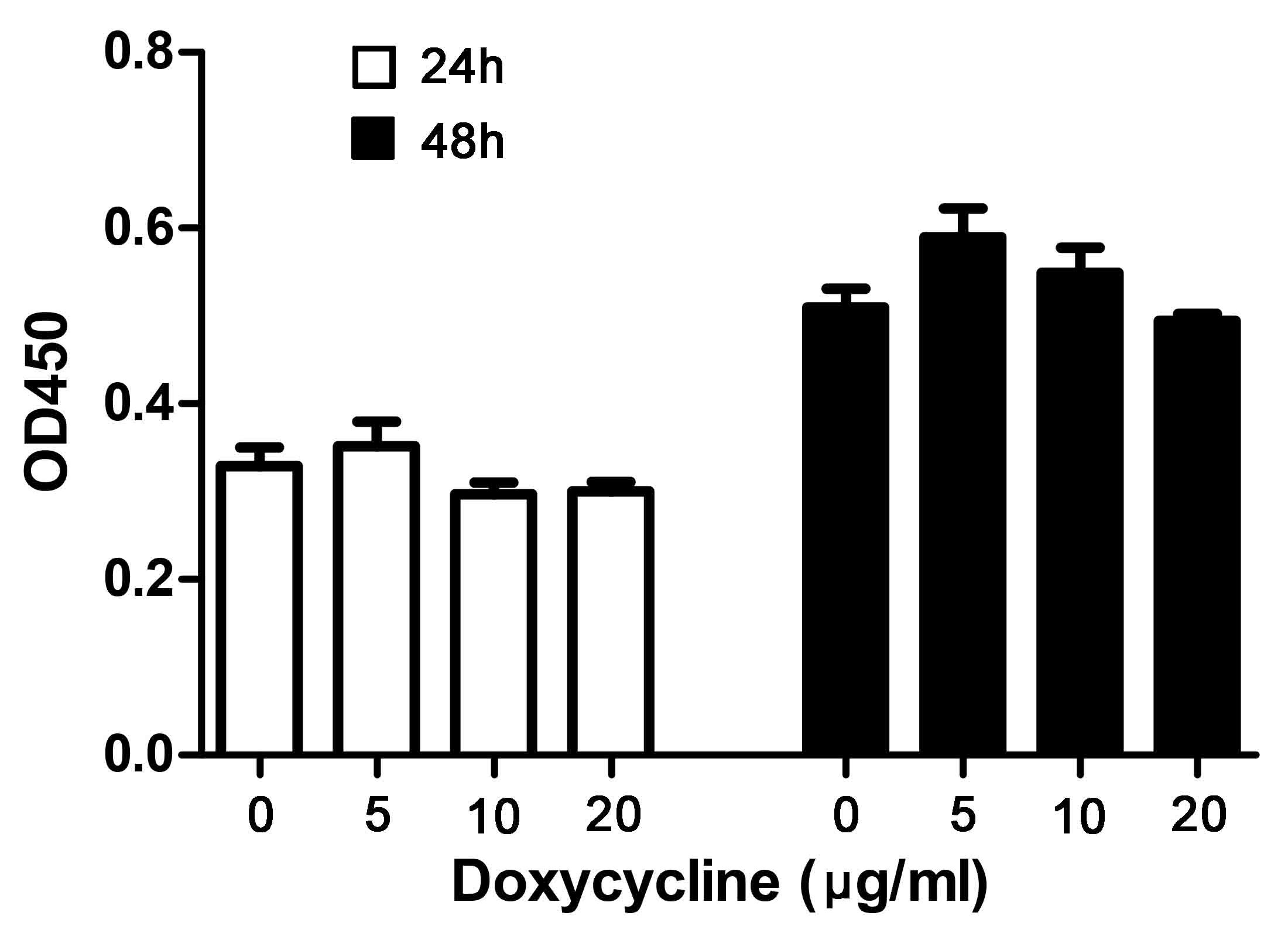

Supplement: Figure S1 — Effect of doxycycline on the cell viability of J774A.1 cells. Cells were cultured with different concentrations of doxycycline (0–20 µg/ml) for 24 and 48 h. The cell viability was determined by CCK-8 kit. Bars show the mean ± SD of three independent experiments. [file image_1.tif]
